# Job autonomy and career development satisfaction among healthcare professionals: the moderating role of organizational identification

**DOI:** 10.3389/fpsyg.2026.1888224

**Published:** 2026-07-13

**Authors:** Yu Xiang, Xueling Guan, Ru Meng, Zhaolin Zhou, Yuxin Qian, Xinru Huang

**Affiliations:** 1 School of Management, Xuzhou Medical University, Xuzhou, China; 2 Institution of Chinese Health Modernization, Xuzhou Medical University, Xuzhou, Jiangsu, China; 3 Medical Insurance Department, Xuzhou Cancer Hospital, Xuzhou, Jiangsu, China

**Keywords:** career development satisfaction, healthcare professionals, job autonomy, nonlinear moderation, organizational identification

## Abstract

**Background:**

Under China’s Healthy China strategy and the high-quality development agenda for public hospitals, healthcare professionals’ career development satisfaction has become critical for service quality and workforce stability. However, most existing studies adopt linear frameworks and fail to capture the nonlinear moderating mechanisms between job autonomy and organizational identification, particularly within the institutional context of Chinese public hospitals.

**Methods:**

Integrating role boundary theory, conservation of resources theory, social exchange theory, and self-determination theory, this study develops an analytical framework to examine how organizational identification moderates the relationship between job autonomy and career development satisfaction. A survey of 398 healthcare professionals from public hospitals was conducted. Multiple linear regression was used to test the main and moderating effects, while quadratic terms were incorporated to examine potential nonlinear relationships. In addition, random forest modeling was applied to assess variable importance and explore complex nonlinear patterns among the study variables.

**Results:**

Job autonomy had a significant positive effect on career development satisfaction and emerged as the strongest predictor. Organizational identification significantly moderated the relationship between job autonomy and career development satisfaction in a nonlinear manner: under low job autonomy, high organizational identification had a compensatory effect; under high job autonomy, it showed an inhibitory effect. Random forest results corroborated the regression findings, confirming the critical role of job autonomy and its interaction with organizational identification.

**Conclusion:**

By identifying the nonlinear moderating role of organizational identification, this study advances understanding of career development satisfaction among healthcare professionals. The combined use of regression and random forest analyses captures both linear and nonlinear relationships. Practically, hospitals should adopt differentiated management strategies by strengthening organizational support for employees with low job autonomy while avoiding excessive conformity pressures on highly autonomous staff, thereby enhancing career development satisfaction and workforce stability.

## 1 Background

Against the dual backdrop of the in-depth implementation of the Healthy China strategy and the precise execution of high-quality development policies for public hospitals, healthcare professionals’ career development satisfaction has become the core indicator for measuring the efficiency of the healthcare service system and ensuring medical quality and patient safety (General Office of the State Council, 2021). With the issuance of a series of policy documents such as the Opinions on Promoting the High-Quality Development of Public Hospitals, China’s medical human resource governance has entered a phase of systematic restructuring, accompanied by profound changes in the career development environment and aspirations of healthcare professionals. However, the structural contradictions currently facing the healthcare industry are becoming increasingly prominent. Data show that by the end of 2024, the total number of health personnel in China reached 15.78 million, an increase of 542,000 (3.6%) from the previous year, yet the growth rate of average daily patient visits to tertiary hospitals more than doubled that of the healthcare workforce (National Health Commission of the People's Republic of China, 2025). This structural imbalance driven by the dual forces of rising medical demand and constrained human resource supply has left healthcare professionals grappling with high-intensity clinical work while facing the dual pressures of rising burnout rates and increased turnover intention (Vander Weerdt et al., 2026). Healthcare professionals’ career development satisfaction is not only crucial for their individual well-being and career longevity but also directly affects the accessibility, safety, and quality of medical services, thereby constraining the effective implementation of the Healthy China strategy. Within this policy context, public hospitals are increasingly emphasizing job autonomy as a means to enhance professional motivation, while simultaneously relying on organizational identification to maintain workforce stability. However, how these two factors interact to shape career development satisfaction remains underexplored.

Improving career development satisfaction is a complex issue involving multiple levels, including individual, organizational, and institutional factors. In the fields of organizational behavior and occupational psychology, job autonomy, defined as an individual’s perceived control over work processes and decision-making authority, is regarded as a key psychological resource influencing job satisfaction (Klein, 2019). Organizational identification, which reflects employees’ emotional attachment, value alignment, and intention to stay with an organization, has increasingly become a focus of scholarly attention regarding its interactive relationship with job autonomy (Hu et al., 2023). Hajir-Afzali et al. (2025) found in a study on hybrid work settings in South Korea that the effect of job autonomy on organizational identification exhibits an “autonomy paradox.” On one hand, it strengthens commitment by enhancing perceived organizational support, but on the other hand, it may weaken employees’ attachment to the organization by fostering psychological independence. This finding reveals the complexity of their relationship (Hajir-Afzali et al., 2025). Ijarew (2022), based on an empirical study of commercial banks in Ethiopia, also pointed out that job autonomy needs to be effectively translated into organizational commitment through the mediating role of autonomous motivation and the moderating role of organizational culture, further corroborating the multidimensional nature of this interactive mechanism.

Although scholars have explored the factors influencing healthcare professionals’ career development satisfaction from multiple perspectives, several gaps remain in the existing literature (Zhao et al., 2024). First, most studies are based on linear analytical frameworks and fail to fully capture the potential nonlinear moderating relationship between job autonomy and organizational identification. Second, some studies directly adopt organizational support scales or related instruments developed in Western contexts, overlooking the institutional and cultural characteristics of China’s public hospital system (Wu et al., 2026). Third, the existing literature pays insufficient attention to the distinct subgroup characterized by “high pressure yet high satisfaction” and lacks in-depth analysis of the underlying mechanisms (Zhu et al., 2024). Moreover, most related studies are grounded in a single theoretical perspective and have yet to effectively integrate multiple analytical frameworks, such as role boundary theory, conservation of resources theory, and social exchange theory (Ji and Cui, 2021; Dai et al., 2022). Against this background, as China’s public hospitals enter a critical phase of institutional reform and digital transformation, the role pressures faced by healthcare professionals are increasingly multilevel and complex. There is an urgent need to adopt an integrated theoretical perspective to systematically investigate the dynamic relationships among job autonomy, organizational identification, and career development satisfaction, as well as their underlying mechanisms.

Based on this, grounded in the Chinese context of public hospitals and integrating role boundary theory, conservation of resources theory, and social exchange theory, this study focuses on the effect of job autonomy on healthcare professionals’ career development satisfaction and provides an in-depth analysis of the nonlinear moderating role of organizational identification, aiming to identify pathways for enhancing career development satisfaction. Role boundary theory emphasizes individuals’ cognitive demarcation and behavioral regulation mechanisms between work and family roles (Ashforth et al., 2000). Conservation of resources theory posits that individuals strive to acquire, maintain, and protect valued resources, and that resource loss has a greater impact than equivalent gain (Hobfoll, 1989). Social exchange theory views organizational support as the process of psychological contract negotiation based on the norm of reciprocity (Blau, 2017). The findings of this study not only enrich the theoretical systems of occupational psychology and organizational behavior by offering new perspectives and empirical evidence for related research but also provide decision-making bases and practical references for public hospitals to optimize human resource management strategies, alleviate occupational stress among healthcare professionals, and enhance career development satisfaction, thereby promoting the sustainable development of the healthcare industry and the effective realization of the Healthy China strategy.

## 2 Literature review and theoretical analysis

### 2.1 Literature review

#### 2.1.1 Core connotations and influencing factors of healthcare professionals’ career development satisfaction

Career development satisfaction is a core dimension of career satisfaction, referring to an individual’s subjective evaluation of their career growth trajectory, development opportunities, professional achievements, and value realization (Wassermann et al., 2017). In the healthcare field, existing research has found that salary levels and career development opportunities have a significant positive impact on healthcare professionals’ career development satisfaction (Moon et al., 2025). However, for healthcare professionals, career development satisfaction encompasses not only external factors such as compensation and promotion pathways but is also closely related to intrinsic psychological and organizational environmental factors, including job autonomy, professional identity, and organizational support.

In recent years, research perspectives have gradually shifted from traditional extrinsic motivational factors to the interactive relationship between individual psychological resources and the organizational environment. As the core job characteristic, job autonomy refers to the freedom individuals have in determining when, where, and how to work. It can foster a sense of responsibility in employees’ psychological states, shield them from distress, activate positive work attitudes and behaviors, and thereby enhance career development satisfaction (Lu et al., 2017; Zito et al., 2019). In the healthcare domain, job autonomy for medical staff is reflected in the discretionary space they possess in areas such as treatment plan formulation, work schedule arrangement, and professional skill development. This autonomy enhances their sense of professional control and self-efficacy (van Dorssen-Boog et al., 2022). Thapa et al. (2025) pointed out that when healthcare professionals actively participate in the development and transformation of medical institutions, their job satisfaction improves, and this satisfaction further enables them to provide higher-quality care to patients.

The “double-edged sword” effect of organizational identification has become increasingly evident in recent research (Riketta, 2016). Financial incentives alone may not necessarily improve the professional well-being or career development satisfaction of healthcare professionals, especially in high-stress work environments, where excessive workloads and emotional exhaustion remain widespread (Millar et al., 2017). This phenomenon aligns with the emotional exhaustion hypothesis in affective labor theory, which suggests that material compensation cannot offset the depletion caused by high-intensity emotional investment; instead, it exacerbates burnout through the psychological mechanism of compensation rationalization (Maslach et al., 2001). Furthermore, high-seniority healthcare professionals exhibit a threshold effect in their perception of organizational support. Excessive support beyond the critical point triggers anxiety about the erosion of job autonomy (Deci and Ryan, 2000), a paradox supported by research advocating for “medical professional group” job autonomy.

#### 2.1.2 Research on the relationships among job autonomy, organizational identification, and career development satisfaction

Existing research has reached a basic consensus on the positive relationship between job autonomy and job satisfaction (Letouche and Wille, 2022). However, the nonlinear characteristics of moderating variables and their boundary conditions are becoming a central focus of investigation. Current studies indicate that job autonomy can enhance organizational identification by improving work-life balance (Reza and Anindita, 2021). Nevertheless, most of this research is set in general workplace contexts and focuses on linear gain relationships, with limited exploration of the potential negative “tipping point” of autonomy in specific high-stress industries. Such exploration is particularly important in healthcare, a high-intensity emotional labor sector. Recent cutting-edge research has begun to suggest that high job autonomy may lead healthcare professionals with strong organizational identification to develop an “excessive sense of responsibility,” prompting them to voluntarily take on overloaded tasks, thereby exacerbating role conflict and diminishing satisfaction (Maisonneuve et al., 2025). Furthermore, within the unique organizational environment of Chinese public hospitals (e.g., the bianzhi personnel system), the relationship between job autonomy and identification may exhibit even more complex patterns, representing an important direction for future indigenous research.

### 2.2 Theoretical analysis

#### 2.2.1 Job autonomy and healthcare professionals’ career development satisfaction: a perspective based on self-determination theory

Self-determination theory (SDT) posits that the satisfaction of autonomy needs concerns not merely external “decision-making freedom” but rather individuals’ perceived alignment between their behaviors and intrinsic values, a concept known as “value identification.” This theoretical framework has been applied and extended in organizational research (Klein, 2019). Specifically in the healthcare context, when medical staff’s autonomous decisions are deeply aligned with their professional values and patient welfare, their job satisfaction is more significantly enhanced. Conservation of resources theory suggests that individuals strive to acquire, maintain, and protect valued resources, and that resource loss has a greater impact than equivalent gain. As an important psychological resource, the adequate supply of job autonomy can effectively reduce healthcare professionals’ resource depletion, thereby enhancing career development satisfaction (Cziraki et al., 2020). Scholarly research on job autonomy and psychological well-being further suggests that job autonomy can indirectly enhance satisfaction via a “leisure activity spillover effect.”: the sense of control gained by healthcare professionals at work extends into non-work domains (e.g., autonomously arranging study or family time), creating a positive psychological cycle that subsequently strengthens career development satisfaction (Dong et al., 2025). Based on this, the following hypothesis is proposed:

*H1*: Job autonomy has a significant positive impact on healthcare professionals’ career development satisfaction.

#### 2.2.2 The moderating role of organizational identification: an integrated perspective based on conservation of resources theory and social exchange theory

Recent research on conservation of resources theory indicates that organizational (or goal) commitment and personal resources engage in a mutually reinforcing and investment-based interactive relationship, rather than a unidirectional path where the former simply leads to the latter (Neveu et al., 2024). This “resource integration effect” enables highly committed individuals to accumulate and utilize resources more effectively, translating job autonomy into career achievement (Gagné and Deci, 2005). Gagné and Deci, drawing on self-determination theory, pointed out that job autonomy promotes the internalization of professional values by stimulating individuals’ autonomous motivation, further enhancing their identification with and sense of responsibility toward the organization, thereby exerting a positive influence on organizational identification (Gagné and Deci, 2005). Li et al.’s study on China’s new-generation employees also showed that when organizational support and leadership style align with employees’ psychological needs, the pathway through which job autonomy influences satisfaction via organizational identification becomes more effective, consistent with the core tenet of conservation of resources theory that “resource matching enhances resource utilization efficiency” (Li, 2025).

Social exchange theory views organizational support as a process of negotiating psychological contracts based on norms of reciprocity. Organizational identification, reflecting employees’ emotional attachment and value alignment with the organization, has an interactive relationship with job autonomy that is essentially a dynamic adjustment process of the psychological contract. However, Hajir-Afzali et al.’s research on the “autonomy paradox” cautions that this exchange relationship has boundaries: when job autonomy is high, healthcare professionals may reduce their expectations of reciprocity toward the organization due to heightened psychological independence, thereby weakening the moderating effect of organizational identification (Hajir-Afzali et al., 2025). Previous research indicates that in high-stress healthcare settings, the positive effects of psychological and organizational resources on work outcomes may be diminished. In such contexts, if organizational identification fails to provide emotional support resources in a timely manner, the positive effects of job autonomy may be offset. A study by Liu et al. on healthcare professionals in China’s Grade A tertiary hospitals found that occupational stress weakens emotional identification and reduces the beneficial role of organizational resources in supporting positive work outcomes (Liu et al., 2023). Li’s cross-national study further indicates that high-stress-induced occupational burnout erodes the positive value of job autonomy, whereas the combination of emotional identification and organizational support can replenish emotional resources and sustain the positive effects of autonomy (Li et al., 2022). In the healthcare context, this nonlinear moderation is particularly evident. Under low job autonomy (e.g., relying on AI for treatment planning), high organizational identification prompts the organization to provide additional compensatory resources (e.g., extra training, flexible scheduling) through emotional ties, mitigating the negative impact of autonomy deficiency. Under high job autonomy (e.g., fully autonomous management of special cases), high organizational identification may lead healthcare professionals to worry about “decision-making errors affecting organizational reputation,” thereby generating anxiety and diminishing satisfaction (Maisonneuve et al., 2025). Based on this, the following hypotheses are proposed:

*H2*: Organizational identification moderates the relationship between job autonomy and healthcare professionals’ career development satisfaction.

*H3*: This moderating effect is nonlinear: organizational identification positively moderates the relationship when job autonomy is low, but negatively moderates it when job autonomy is high.

**Figure 1:**
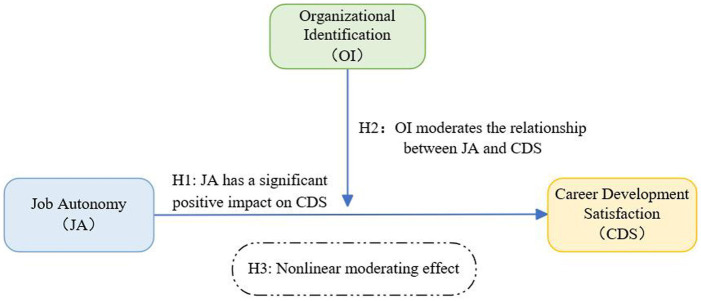
Hypothesis framework.

Based on the hypotheses proposed above, the research hypothesis framework is presented in Figure 1.

**Figure 2:**
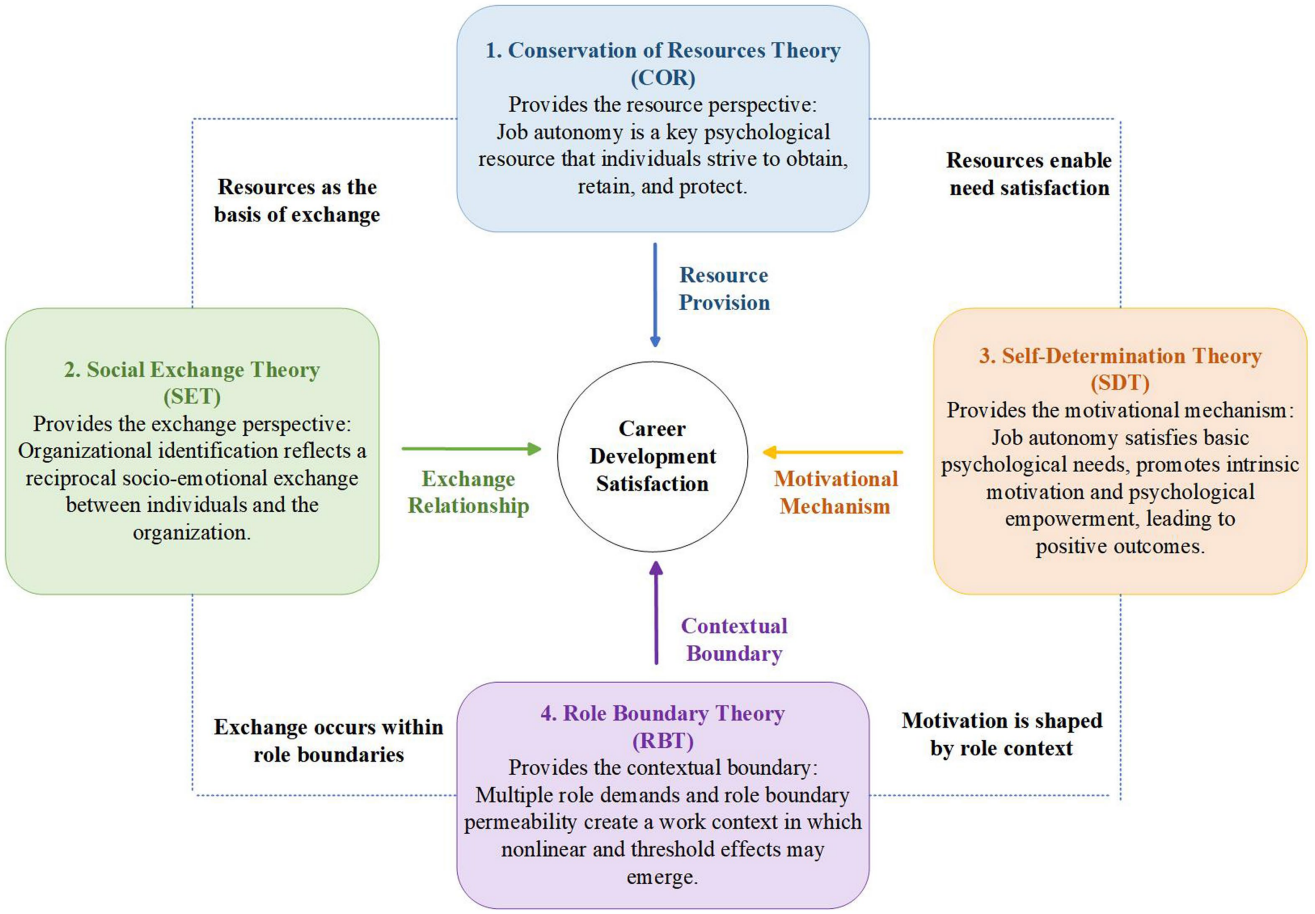
Diagram of the theoretical integration framework.

To integrate multiple theoretical perspectives, this study constructs a unified hierarchical framework to explain how job autonomy and organizational identification jointly influence career development satisfaction (Figure 2). Based on conservation of resources (COR) theory (Hobfoll, 1989), job autonomy is conceptualized as a key personal resource that enables the acquisition and maintenance of resources, thereby initiating a virtuous cycle that enhances an individual’s capacity for sustained career development. Building on this, social exchange theory posits that organizational identification reflects the quality of reciprocal socio-emotional exchanges between employees and the organization (Blau, 2017); positive exchange relationships reinforce organizational identification and enhance positive work-related evaluations. Building on these structural foundations, self-determination theory (Deci and Ryan, 2000) elucidates the internal psychological mechanisms through which job autonomy promotes the fulfillment of basic psychological needs, thereby stimulating intrinsic motivation and psychological internalization, and translating resource gains and exchange quality into higher career development satisfaction. Finally, role boundary theory (Ashforth et al., 2000) describes the contextual conditions in which these processes operate, positing that in complex professional environments such as healthcare, multiple and overlapping role demands lead to variations in boundary permeability, thereby shaping the strength of the relationship between job autonomy, organizational identification, and career outcomes, as well as its potential nonlinear characteristics. Overall, these theories are not mutually exclusive explanatory perspectives but are integrated hierarchically: Conservation of resources theory and social exchange theory constitute the structural foundation at the macro level; self-determination theory clarifies the motivational transmission processes at the micro level; and role boundary theory defines the situational boundary conditions. Together, they determine career development satisfaction.

## 3 Method

### 3.1 Study design

This study used a cross-sectional survey design.

### 3.2 Participants

This study employed convenience sampling to recruit clinical healthcare professionals from multiple public hospitals in Xuzhou, Jiangsu Province, from May to June 2025. Xuzhou was selected as the study area because it serves as the major regional medical hub in northern Jiangsu, home to several large public hospitals of various types that bring together a diverse pool of healthcare professionals. The hospitals participating in the study differ in terms of size, departmental composition, and scope of services, providing an appropriate research context for examining the relationship among job autonomy, organizational identification, and career development satisfaction. Furthermore, the accessibility of these hospitals facilitates data collection and ensures a sufficient sample size for empirical analysis. According to Kendall’s sample size estimation method, the sample size should be 10–20 times the number of items in the measurement instrument (Sen, 1968). The questionnaire used in this study contains 11 items, including 3 items on job autonomy, 3 items on organizational identification, and 5 items on career development satisfaction. Therefore, the recommended sample size range is 110 to 220. Taking into account a 20% non-response rate, the minimum required sample size is 138.

Inclusion criteria: (1) Possession of nationally certified professional qualifications (physician, nurse, pharmacist, technician, etc.); (2) Active frontline clinical staff; (3) Informed about the study and willing to participate. Exclusion criteria: (1) Interns, trainees, or residents; (2) Those on extended leave, attending off-site training, or in non-clinical positions during the survey period.

The survey was distributed via the Wenjuanxing online platform. A total of 423 questionnaires were collected. After excluding 25 invalid responses (due to incomplete answers or obvious errors), the final sample size was 398, yielding a valid response rate of 94.1%. The final sample size met and exceeded the minimum requirement. The participants in this study were recruited from several public hospitals in Xuzhou, Jiangsu Province; therefore, the sample primarily reflects the characteristics of healthcare personnel in public hospitals in that region. In addition, medical technicians constitute a relatively high proportion of the sample, while nurses, clinicians, and primary healthcare workers account for a smaller proportion. Consequently, the characteristics of the sample composition should be fully taken into account when interpreting and generalizing the study findings.

### 3.3 Instruments

#### 3.3.1 Demographic characteristics

This self-designed questionnaire collected demographic data, including age, marital status, number of children, educational attainment, monthly household income, technical field, and years of working.

#### 3.3.2 Job autonomy scale (JAS)

Job autonomy was measured using the classic scale developed by Hackman and Oldham (1975) in their Job Characteristics Model. This study selected 3 items measuring the core dimension of “autonomy,” including: “The job allows me to make my own decisions about how to schedule my work,” “The job gives me a chance to use my personal initiative or judgment in carrying out the work,” “The job gives me considerable opportunity for independence and freedom in how I do the work.” These items were rated on a 5-point Likert scale (1 = “strongly disagree,” 5 = “strongly agree”). The total score ranges from 3 to 15, with higher scores indicating greater perceived job autonomy among employees. This scale is widely used in organizational behavior research, with a Cronbach’s *α* of 0.963 for this dimension in the present study.

#### 3.3.3 Organizational identification scale (OIS)

Measurement was conducted using three items from the organizational identification scale developed by Mael and Ashforth (1992). The selected items are as follows: “When I talk about this hospital, I usually say ‘we’ rather than ‘they’” “This hospital’s successes are my success” “When someone praises this hospital, it feels like a personal compliment.” These items were rated on a 5-point Likert scale (1 = “strongly disagree,” 5 = “strongly agree”). The total score ranges from 3 to 15, with higher scores indicating stronger organizational identification among employees toward the hospital. This scale effectively reflects the cognitive and affective unity between individuals and organizations. In this study, the Cronbach’s *α* for this dimension was 0.877.

#### 3.3.4 Career satisfaction scale (CSS)

Career development satisfaction refers to an individual’s subjective evaluation of their career growth trajectory, development opportunities, career achievements, and the realization of career values (Wassermann et al., 2017). To measure this construct, this study employed the Career Satisfaction Scale (CSS) developed by Greenhaus et al. (1990). Although this scale was originally designed to assess career satisfaction, its five items comprehensively cover the key dimensions of career development satisfaction. This study utilized all five items: “I am satisfied with the success I have achieved in my career,” “I am satisfied with my progress toward achieving my overall career goals,” “I am satisfied with my progress toward achieving my income goals,” “I am satisfied with my progress toward achieving my promotion goals,” and “I am satisfied with my progress toward achieving my goals for developing new skills.” These items were rated using a 5-point Likert scale (1 = “Strongly Disagree,” 5 = “Strongly Agree”), with the total score ranging from 5 to 25; a higher score indicates greater satisfaction. Satisfaction with overall career goals and with the development of new skills directly reflects an individual’s perception of career growth and development; satisfaction with promotion and income represents important external indicators and outcomes of career development; and satisfaction with career success reflects an overall evaluation of career achievements and progress. Therefore, the content covered by the CSS aligns closely with the conceptual domains of career development satisfaction adopted in this study. The CSS is one of the most widely used and empirically validated scales in the field of career research (Greenhaus et al., 1990), with its reliability and validity supported across various occupational contexts. In this study, its Cronbach’s alpha coefficient was 0.765, indicating acceptable internal consistency. Furthermore, previous studies have employed the CSS to operationalize individuals’ satisfaction with career development and career progress (Li et al., 2025). Therefore, this study employs the CSS as an appropriate measurement tool for assessing career development satisfaction among healthcare professionals.

The internal consistency reliability of each scale was assessed using Cronbach’s *α*. According to the recommendations of Hair et al. (2010), a Cronbach’s α value above 0.70 indicates acceptable internal consistency reliability. All three scales exceeded the recommended threshold (minimum 0.765), indicating satisfactory internal consistency.

### 3.4 Data collection and ethical considerations

This study used the Wenjuanxing online platform to create an electronic version of a structured questionnaire. The questionnaire included detailed information on the study’s objectives, main content, and academic significance, along with instructions for completion. It also included an informed consent statement emphasizing that participation was entirely voluntary, that all responses would be anonymous and strictly confidential (used solely for academic research), and that participants had the right to withdraw unconditionally at any time without incurring any adverse consequences. The questionnaire content was reviewed by the research team to exclude questions that might cause psychological distress or harm, and submissions were limited to one per IP address to ensure data quality. After obtaining informed consent from the relevant administrative departments and clinical departments at the target hospital, the researchers distributed the link to the electronic questionnaire via the hospital’s internal work communication group. Eligible healthcare professionals voluntarily and independently completed the questionnaire during work breaks or in their personal time after reading and agreeing to the informed consent form. The entire online data collection process lasted approximately 2 months. This study adhered to the ethical principles of the Declaration of Helsinki and received approval from the Institutional Ethics Committee of Xuzhou Medical University prior to data collection. All participants provided informed consent electronically; data were collected anonymously and used solely for research purposes.

### 3.5 Statistical analyses

This study utilized SPSS 26 and MATLAB for data analysis; the two methods serve distinct yet complementary research objectives. First, SPSS 26 was used to conduct reliability and validity tests for the scales, descriptive statistics, correlation analysis, and multiple regression analysis. Prior to conducting the regression analysis, to test for potential common-method bias in the cross-sectional questionnaire data, this study performed a supplementary analysis using Harman’s one-factor test. Regression analysis was primarily used to examine the effects of job autonomy, organizational identification, and their interaction on career development satisfaction, and to test the research hypotheses while controlling for demographic variables.

Specifically, the following regression equations were estimated (Equation 1):


$$\mathrm{CDS}=\beta_{0}+\beta_{1}\;\mathrm{JA}+\sum \beta_{k}\text{Controls}+\varepsilon$$ (1)

Moderation Effect Model (Equation 2):


$$\mathrm{CDS}=\beta_{0}+\beta_{1}\;\mathrm{JA}+\beta_{2}\;\mathrm{OI}+\beta_{3}(\mathrm{JA}\times \mathrm{OI})+\sum \beta_{k}\;\text{Controls}+\varepsilon$$ (2)

In this context, *CDS* represents career development satisfaction, *JA* represents job autonomy, *OI* represents organizational identification, *Controls* represents demographic control variables, *β* represents the regression coefficient, and ε represents the random error term.

Quadratic terms were introduced into the models to test for nonlinear moderating effects. Second, the random forest model was constructed using MATLAB. Unlike traditional linear regression, random forests can identify potential nonlinear relationships and complex interaction patterns among variables; therefore, they were used to assess the importance of each predictor variable and to further illustrate the nonlinear influence of job autonomy and organizational identification on career development satisfaction through partial dependence plots. The two methods are complementary. Multiple linear regression is primarily used for theory-driven hypothesis testing and the estimation of net effects, while random forest analysis is primarily used for assessing variable importance and exploring nonlinear relationships. By combining traditional statistical methods with machine learning methods, this study is able to validate the research results from different perspectives, thereby enhancing the robustness and interpretability of the conclusions.

## 4 Results

### 4.1 Demographic information

When the demographic information of healthcare professionals was analyzed, it was found that the 40–49 age group constitutes the largest proportion (42.96%), followed by the 30–39 age group (30.90%). The proportion of those aged 50 and above is relatively low (11.31%). Marital status was predominantly married (89.20%), with relatively fewer divorced (6.78%) and widowed (3.02%) individuals. Monthly household income showed significant variation, with 57.79% earning over ¥60,000 monthly, while only 6.53% earned ¥10,000 or less. The number of children per household predominantly featured two children (50.25%), with households having four or more children accounting for nearly 20%. In terms of years of working, those with over 10 years of experience dominated (40.95% had 10–15 years, and 35.68% had 16 years or more). Regarding technical fields, medical technology professionals constituted 71.11%, significantly higher than nursing (9.30%) and public health and management (17.34%). Overall data indicates that survey respondents possess high professional qualifications, extensive career experience, and notable economic characteristics (Table 1).

**Table 1:** Demographic information of healthcare professionals (*n* = 398).

Socio-demographic characteristics	*n*	%
Age		
30 and under	59	14.8
30–39 years old	123	30.9
40–49 years old	171	43
50 years and older	45	11.3
Marital status		
Unmarried	4	1
Married	355	89.2
Divorced	27	6.8
Widowed	12	3
Educational attainment		
High school and below	10	2.5
Associate degree	46	11.6
Undergraduate	181	45.5
Postgraduate and above	161	40.5
Monthly household income		
10,000 and below	26	6.5
10,001–30,000	67	16.8
30,001-60,000	75	18.8
60,000 and above	230	57.8
Number of children		
1 or fewer	66	16.6
2	200	50.3
3	53	13.3
4 or more	79	19.8
Years of working		
3 years or less	14	3.5
4–9 years	79	19.8
10–15 years	163	41
16 years or more	142	35.7
Technical field		
Clinical medicine	9	2.3
Nursing	37	9.3
Medical technology	283	71.1
Public health and management	69	17.3

n: Sample, %: percentage.

### 4.2 Evaluation of the measurement model

To assess the reliability and validity of the measurement model, this study conducted confirmatory factor analysis (CFA). The results indicated that the measurement model had acceptable fit indices (χ^2^/df = 3.181, CFI = 0.972, GFI = 0.959, RMSEA = 0.074, RMR = 0.049), suggesting that the overall model fit was satisfactory.

As shown in Table 2, all standardized factor loadings were above 0.80, indicating high item reliability and adequate construct validity. Furthermore, the composite reliability (CR) values for all constructs exceeded 0.70, demonstrating good internal consistency. The average variance extracted (AVE) values for each construct were all above 0.50, indicating satisfactory convergent validity. Discriminant validity was also verified; the square root of the AVE for each construct was greater than the correlation coefficient between constructs, confirming sufficient discrimination among the variables. The internal consistency of each subscale was assessed using Cronbach’s alpha (*α*), which reflects the homogeneity of items within the subscale. The minimum structural reliability coefficient was 0.765, exceeding the acceptance criterion of 0.6 (Kim and Karatepe, 2023). All Cronbach’s alpha coefficients were above the reliability standard.

**Table 2:** Results of measurement model validation.

Concept	Measurement items	Standardized factor loadings	AVE	CR
Job autonomy (JA)	JA1	0.828	0.700	0.875
	JA2	0.812		
	JA3	0.868		
Organizational identification (OI)	OI1	0.889	0.774	0.911
	OI2	0.868		
	OI3	0.883		
Career development satisfaction (CDS)	CDS1	0.839	0.704	0.922
	CDS2	0.850		
	CDS3	0.885		
	CDS4	0.813		
	CDS5	0.802		

### 4.3 Common method bias test

Since this study collected data through the cross-sectional questionnaire, common method bias may be present. To assess the extent of this common method bias, we employed a one-factor test based on the proportion of variance explained by the Harman subfactor in the unrotated solution. The results showed that the first unrotated factor explained 34.18% of the total variance, which is below the recommended threshold of 40%. This indicates that there is no serious common method bias in this study (Zhou and Long, 2004).

### 4.4 Multiple linear regression analysis

To test the research hypotheses, this study constructed a series of multiple linear regression models (Tables 3–5). First, the full-variable model results shown in Table 3 indicate that job autonomy (JA) is the strongest predictor of career satisfaction (*β* = 0.474, *p* < 0.001), with its standardized coefficient significantly higher than other variables. According to this result, hypothesis H1 is accepted. Age (*β* = 0.14, *p* = 0.002) and educational attainment (*β* = 0.11, *p* = 0.011) exerted significant positive effects on career satisfaction, whereas monthly household income demonstrated the significant negative impact (*β* = −0.14, *p* = 0.003). Notably, none of the technical field categorical variables (clinical medicine, nursing, public health and management) reached statistical significance (*p* > 0.05), and organizational identification (OI) also showed no significant effect (*β* = 0.05, *p* = 0.250). The variance inflation factor (VIF) for all independent variables was well below 10, indicating no multicollinearity issues in the model (Gujarati, 2003). Among the technical field variables, the medical technology category had zero tolerance, indicating perfect collinearity with other variables. This category was therefore set as the reference group, and its parameter could not be estimated independently. The remaining technical field categories (clinical medicine, nursing, public health and management) were entered as dummy variables in the regression model.

**Table 3:** Multiple linear regression model with all variables.

Variables	Unstandardized Coefficient		Standardized Coefficient	*t*	*p*-value	Correlation			Collinearity Statistics	
	B	SE	Beta			Zero-order	Partial	Part	Tolerance	VIF
Intercept	1.02	0.44		2.34	0.020					
Age	0.15	0.05	0.14**	3.19	0.002	0.17	0.16	0.13	0.85	1.18
Marital status	0.08	0.13	0.03	0.64	0.524	0.03	0.03	0.03	0.96	1.04
Educational attainment	0.14	0.05	0.11*	2.56	0.011	0.14	0.13	0.11	0.87	1.15
Monthly household income	−0.13	0.04	−0.14**	−2.94	0.003	−0.11	−0.15	−0.12	0.83	1.21
Number of children	−0.07	0.04	−0.07	−1.53	0.126	−0.10	−0.08	−0.06	0.80	1.25
Years of working	0.07	0.05	0.06	1.36	0.174	0.06	0.07	0.06	0.85	1.18
Technical field 1	0.10	0.26	0.02	0.38	0.701	0.04	0.02	0.02	0.98	1.02
Technical field 2	−0.03	0.14	−0.01	−0.24	0.814	−0.04	−0.01	−0.01	0.93	1.07
Technical field 3	−0.04	0.10	−0.02	−0.42	0.674	−0.07	−0.02	−0.02	0.95	1.05
JA	0.91	0.09	0.47***	10.78	0.000	0.53	0.48	0.45	0.89	1.12
OI	0.03	0.03	0.05	1.15	0.250	0.19	0.06	0.05	0.92	1.09

***p < 0.001; **p < 0.01; *p < 0.05; SE: Standard Error; VIF: Variance Inflation Factor; JA: Job autonomy; OI: Organizational identification; Technical field 1: Clinical medicine; Technical field 2: Nursing; Technical field 3: Public health and management.

**Table 4:** Simplified linear regression model results (Job autonomy and organizational identification).

Variables	Unstandardized coefficient		Standardized coefficient	*t*	*p*-value	Correlation			Collinearity statistics	
	*B*	SE	Beta			Zero-order	Partial	Part	Tolerance	VIF
Intercept	1.48	0.12		12.05	0.000					
JA	0.99	0.08	0.52***	11.87	0.000	0.53	0.51	0.50	0.95	1.06
OI	0.05	0.03	0.07	1.69	0.091	0.19	0.09	0.07	0.95	1.06

***p < 0.001; SE: Standard Error; VIF: Variance Inflation Factor; JA: Job autonomy; OI: Organizational identification.

**Table 5:** Results of the moderated effect model.

Variables	Unstandardized Coefficient		Standardized Coefficient	t	*p*-value	Correlation			Collinearity Statistics	
	B	SE	Beta			Zero-order	Partial	Part	Tolerance	VIF
Intercept	1.30	0.14		9.55	0.000					
JA	1.10	0.09	0.57***	12.21	0.000	0.53	0.52	0.51	0.81	1.24
OI	0.05	0.03	0.09	1.96	0.051	0.19	0.10	0.08	0.94	1.06
JA × OI	−0.12	0.04	−0.14**	−2.97	0.003	0.11	−0.15	−0.13	0.83	1.21

***p < 0.001; **p < 0.01; SE: Standard Error; VIF: Variance Inflation Factor; JA: Job autonomy; OI: Organizational identification.

The simplified model focuses on core variables (Table 4), where job autonomy remained highly significant (*β* = 0.517, *p* < 0.001), while organizational identification approached the critical threshold (*β* = 0.07, *p* = 0.091). After model adjustment, the explanatory power of job autonomy further increased, with the standardized coefficient rising by approximately 9.0% compared to the full-variable model.

After introducing the interaction term between job autonomy and organizational identification (Table 5), the moderation effect model revealed significant results. The interaction coefficient was negative and statistically significant (*β* = −0.14, *p* = 0.003), indicating that organizational identification moderated the relationship between job autonomy and career satisfaction. Hypothesis H2 is accepted. Specifically, at high levels of organizational identification, the positive impact of job autonomy on satisfaction was attenuated. Hypothesis H3 is accepted. At this point, the main effect of organizational identification became marginally significant (*β* = 0.09, *p* = 0.051), while job autonomy remained the strongest predictor (*β* = 0.571, *p* < 0.001). Tolerance indices (>0.80) and VIF values (<1.24) for all models satisfied multicollinearity assumptions, confirming the robustness of the model construction.

### 4.5 Moderation effect analysis

**Figure 3:**
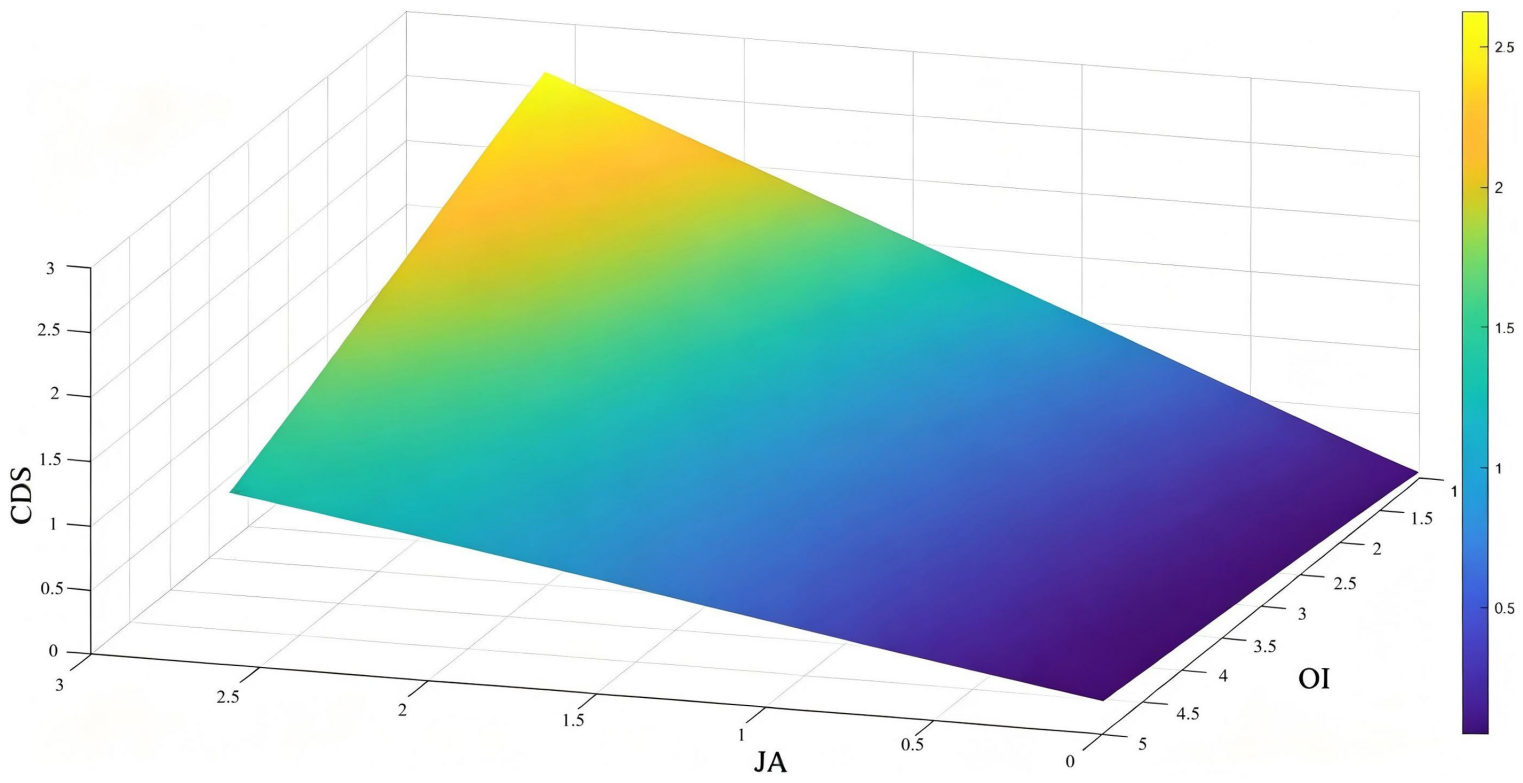
Interaction effect surfaceplot.

The results of the moderating effects analysis (Table 5) indicate that the interaction term between job autonomy and organizational identification has a significant negative effect on career development satisfaction (*β* = −0.14, *p* = 0.003), suggesting that organizational identification significantly moderates the relationship between job autonomy and career development satisfaction (H2). The three-dimensional interaction effect surface plot based on the regression model was generated using MATLAB (Figure 3) to visually illustrate the combined influence of job autonomy and organizational identification on career development satisfaction.

To further examine whether this moderating effect exhibits nonlinear characteristics, this study estimated a quadratic model by introducing the squared term of job autonomy (JA^2^) and its interaction term with organizational identification (JA^2^ × OI) into the regression model. The results showed that the squared term of job autonomy was statistically significant and negative (*β* = −0.4685, *p* = 0.0016), indicating a curvilinear relationship (inverted U-shaped relationship) between job autonomy and career development satisfaction. More importantly, the interaction term between JA^2^ and organizational identification was also statistically significant (*β* = −0.2221, *p* = 0.0177), indicating that organizational identification not only affects the strength of the relationship between job autonomy and career development satisfaction but also influences its curvature; that is, organizational identification exerts a significant nonlinear moderating effect on this relationship. Therefore, the results of the quadratic term provide statistical support for H3. The moderating effect of organizational identification is nonlinear, indicating that its influence on the relationship between job autonomy and career satisfaction varies across different levels of job autonomy.

### 4.6 Random forest

**Figure 4:**
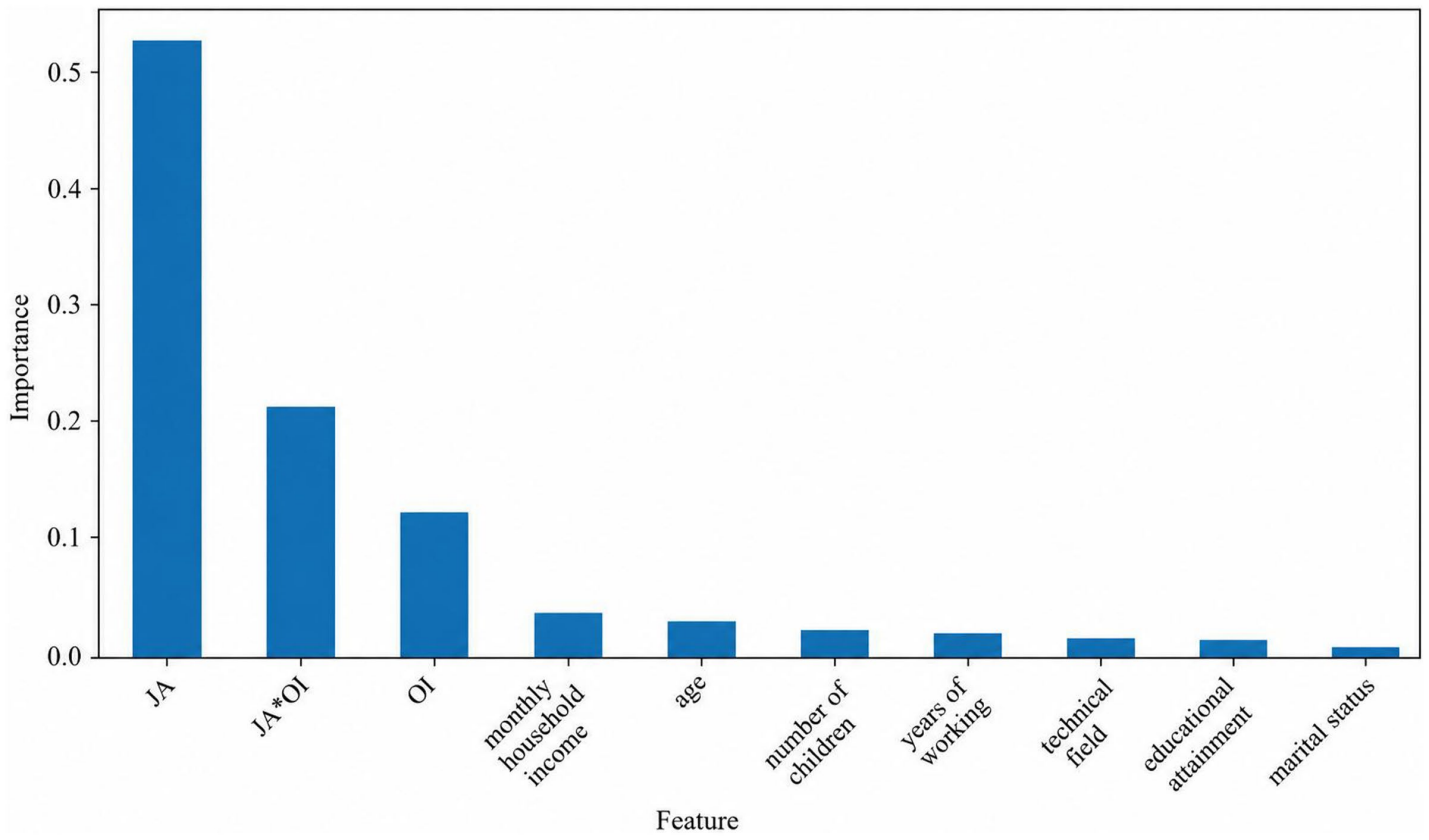
Random forest feature importance ranking.

**Figure 5:**
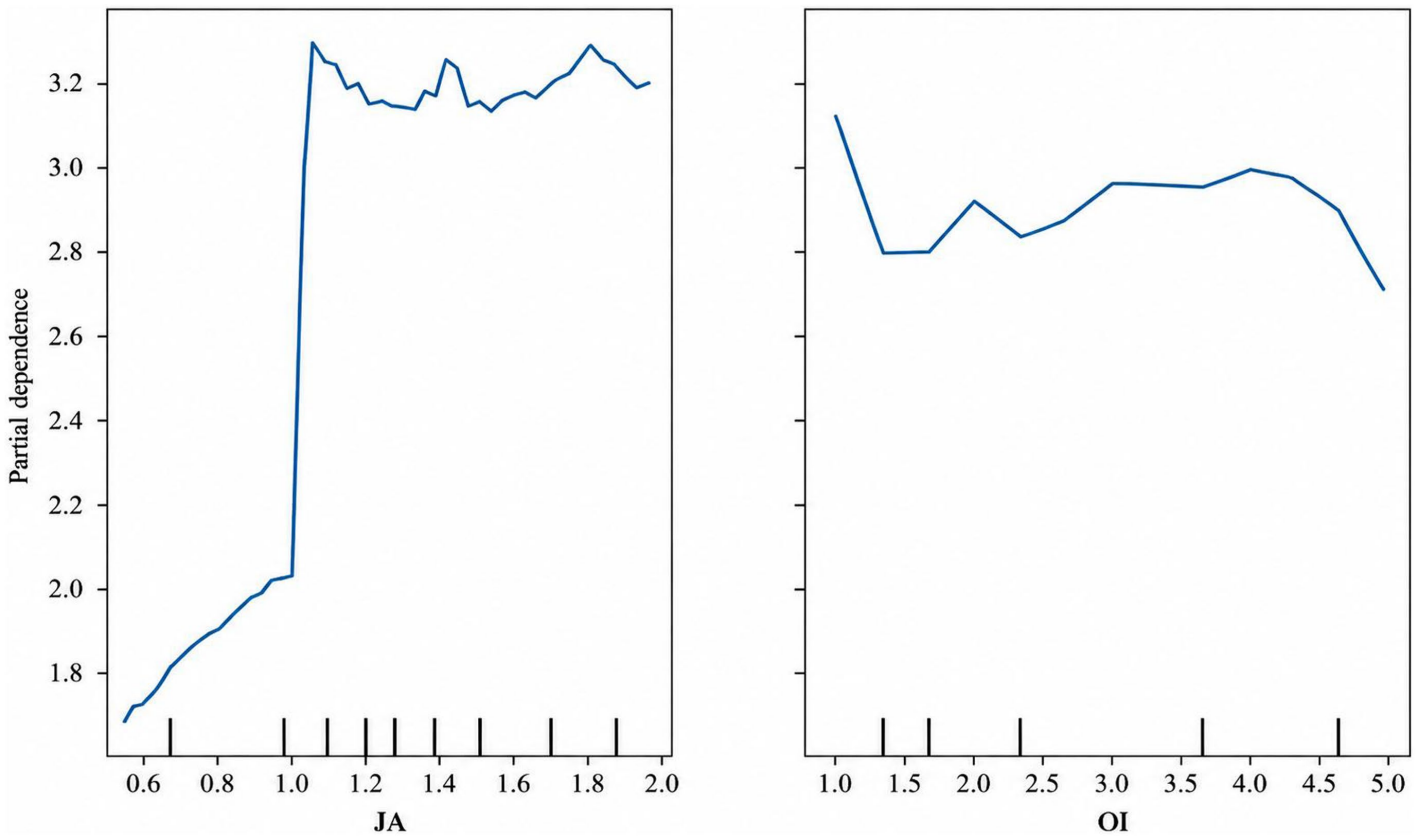
Partial dependency diagram of JA and OI.

**Figure 6:**
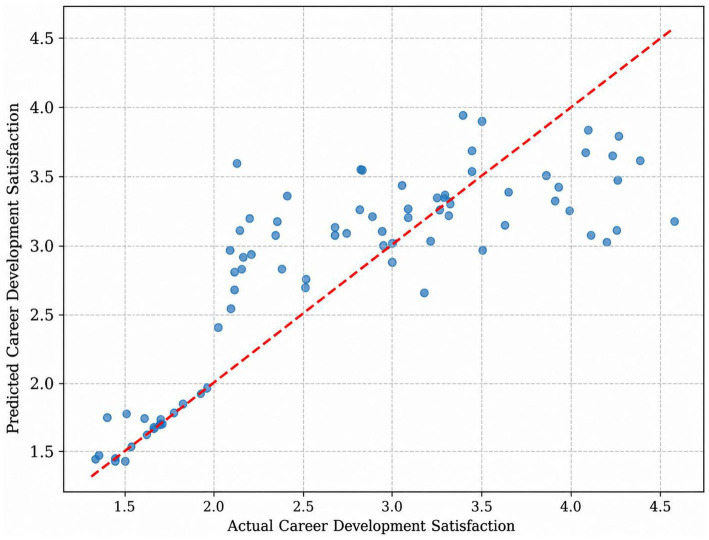
Prediction of career development satisfaction.

This study employed a random forest regression model to assess the importance of various factors influencing healthcare professionals’ career development satisfaction and to explore potential nonlinear and interactive relationships among the variables. Figures 4–6 illustrate the ranking of variable importance, interaction patterns, and the model’s predictive performance, respectively.

As shown in Figure 4, job autonomy (JA) received the highest feature importance score, significantly exceeding all other predictor variables and accounting for more than 50% of the total importance contribution. This indicates that, within the Random Forest framework, JA is the most important predictor of career development satisfaction. The interaction term between job autonomy (JA) and organizational identification (OI) ranked second in importance, surpassing even the main effect of organizational identification, suggesting that the combined information of these two variables contributes more to the prediction than organizational identification (OI) alone. In contrast, demographic variables (e.g., age, marital status) and socioeconomic factors (e.g., monthly household income) had lower importance scores, suggesting that their predictive contribution is limited once psychological variables are included.

To further explore how job autonomy and organizational identification jointly influence career development satisfaction, this study used partial dependency plots (Figure 5) to visualize the model’s predictive response patterns. The results show that career development satisfaction increases with greater job autonomy, but the magnitude of this increase varies across different levels of organizational identification. Specifically, at higher levels of organizational identification, the predicted values of job satisfaction are generally higher, and they exhibit a more pronounced response to changes in job autonomy; at lower levels of organizational identification, the predicted values of job satisfaction are relatively lower, and the response to job autonomy is also relatively weaker. It should be noted that organizational identification was treated as a continuous variable throughout this study; the terms “higher” and “lower” organizational identification used here refer only to the relatively high and relatively low ranges of organizational identification observations within the distribution shown in the figure. Overall, this suggests that the combination of organizational identification and job autonomy influences the magnitude of the predicted career development satisfaction but does not alter its fundamental direction of change.

It is crucial to clarify the relationship between the regression results and the random forest results. The multiple linear regression model, under a parametric framework, estimated the average net moderating effect of organizational identification on the relationship between job autonomy and career development satisfaction after controlling for relevant covariates; this result reflects the overall directional effect at the full-sample level within a linear framework. In contrast, the random forest model offers a non-parametric, data-driven perspective that emphasizes predictive performance and captures nonlinear and heterogeneous response patterns. Partial dependency plots illustrate the structural variations in predictive outcomes across different variable combinations, rather than traditional interaction coefficients or directional effects. Consequently, these two methods serve distinct analytical purposes: regression models are used to test hypotheses and estimate average effects, while random forests are used to characterize complex nonlinear predictive structures. Consequently, the two should not be viewed as contradictory but rather as complementary characterizations of the same relationship.

Figure 6 shows a scatter plot of job satisfaction predicted by the Random Forest model against observed values. The vast majority of observations are tightly clustered around the reference line, particularly in the higher satisfaction ranges, indicating that the model exhibits good predictive accuracy. Some dispersion is observed in the low-to-medium ranges, which may reflect individual heterogeneity or unobserved factors that the model failed to capture. Overall, the model demonstrates satisfactory predictive performance and robustness.

## 5 Discussion

This study, grounded in the role boundary perspective, specifically analyzed the impact of job autonomy, organizational identification, monthly household income, age, number of children, years of service, technical field, educational attainment, and marital status on career satisfaction. Results indicate that career satisfaction can be largely predicted by the combined influence of job autonomy and organizational identification.

### 5.1 Analysis of factors influencing career satisfaction among healthcare professionals

Job autonomy (*β* = 0.474, *p* < 0.001) emerged as a key determinant of career development satisfaction among healthcare professionals. This finding aligns closely with the theory proposed by Edward in 2017 (Deci et al., 2017), indicating that when healthcare professionals can independently decide their work methods, pace, and content, their satisfaction with professional growth significantly increases (Delic et al., 2021). Consistent with prior research (Romadhona and Murti, 2024), this study found that age (*β* = 0.14, *p* = 0.002) and educational attainment (*β* = 0.11, *p* = 0.011) positively influence healthcare professionals’ career satisfaction. This suggests that senior healthcare professionals may achieve higher career development satisfaction due to accumulated work experience and a more proactive work attitude (Medeni et al., 2025), while those with higher educational attainment may attain greater satisfaction through increased social prestige, authority, and job autonomy (Burney et al., 2023). Family economic status (*β* = −0.14, *p* = 0.003) exerted a negative influence on healthcare professionals’ career development satisfaction. This finding contradicts the prevailing conclusions of related studies (Jeon et al., 2024; Li et al., 2023). It is speculated that for healthcare professionals from high-income families, higher income not only fails to significantly enhance their well-being but may even reduce it due to increased pressure (Diener and Biswas-Diener, 2022), thereby diminishing career development satisfaction. One plausible explanation is that high-income healthcare professionals may hold higher expectations for career growth and work–life balance; when these expectations are not met by the rigid structure of public hospital systems, the positive effect of income on satisfaction may be offset. However, this interpretation requires further empirical validation, as the present study did not directly measure perceived pressure or expectation levels.

For them, freedom in life choices may be the dominant factor in enhancing well-being (Behera et al., 2024). However, the unique nature of the healthcare profession often prevents these needs from being met (Gökhan et al., 2024), resulting in a negative correlation between household economic conditions and career development satisfaction. In this study, marital status, number of children, years of service, and specific medical technical fields did not show a significant impact on career development satisfaction. Additionally, the experiment concluded that organizational identification has a relatively weak direct influence on healthcare workers’ career development satisfaction and should be considered in conjunction with job autonomy. According to John P. Meyer’s three-factor theory of organizational identification (Meyer and Allen, 1991), this may be the combined result of these three factors exerting different influences on career development satisfaction (Meyer et al., 2002).

### 5.2 Analysis of the interactive effects on healthcare professionals’ career development satisfaction

The results of this study indicate an interaction effect between job autonomy and organizational identification (*β* = −0.14, *p* = 0.003), suggesting that job autonomy exerts significant compensatory and inhibitory effects on organizational identification. This finding aligns with Self-Determination Theory’s discussion on the internalization of extrinsic motivation (Deci and Ryan, 2000). From the perspective of motivational internalization in self-determination theory, Zhao et al. (2025) confirmed through their study of frontline healthcare professional that when job autonomy fails to satisfy core intrinsic motivational needs, individuals tend to deeply integrate organizational developmental goals with their own professional ethical norms through value alignment. The external manifestation of this integration process is precisely high levels of affective identification and identity identification. This internalized, relatively autonomous form of motivation effectively compensates for the insufficient fulfillment of psychological needs (particularly autonomy needs) caused by the lack of job autonomy. It becomes a key resource driving healthcare workers to seek meaning in monotonous or controlled tasks, persist in professional development, and ultimately achieve satisfaction. More intriguingly, research indicates that at high levels of job autonomy, the promotional effect of organizational identification on career development satisfaction diminishes. This may be understood in terms of motivation’s “quality” rather than “quantity.” According to self-determination theory, highly autonomous work itself can fully satisfy employees’ needs for autonomy and competence, thereby generating strong intrinsic motivation (Bogdán et al., 2024). In such contexts, the presence of extremely high organizational identification, particularly when mixed with introjected regulation components rooted in obligation or guilt, may subtly interfere with or constrain an individual’s pure experience of autonomy. This may shift the motivational focus from “I enjoy and lead this work” to “I must be accountable to the organization,” thereby diluting the peak experiences derived from intrinsic motivation and flattening the slope of satisfaction growth associated with increased autonomy. This suggests that in highly specialized, autonomous medical work, cultivating healthcare professionals’ intrinsic passion for the work itself may be equally important as fostering loyalty and identification to the organization. Both require balance to avoid the latter potentially inhibiting the former.

### 5.3 Analysis of the moderating effects on healthcare professionals’ career development satisfaction

Based on role boundary theory and social exchange theory, organizational identification functions as an important contextual factor that shapes the relationship between job autonomy and healthcare professionals’ career development satisfaction (Ashforth et al., 2000). The present findings indicate that organizational identification significantly moderates the association between job autonomy and career satisfaction, suggesting that the effectiveness of job autonomy depends on employees’ psychological attachment to their organization (Mael and Ashforth, 1992). Specifically, when organizational identification is relatively high, healthcare professionals are more likely to interpret autonomy as a signal of organizational trust and professional recognition. Under such conditions, increases in job autonomy are associated with stronger improvements in career development satisfaction. Strong organizational identification may enhance employees’ willingness to invest effort in their work, strengthen their perception of organizational support, and increase the positive value they derive from autonomous work experiences (Stinglhamber et al., 2015). In contrast, when organizational identification is relatively low, the positive influence of job autonomy on career development satisfaction becomes weaker. Although autonomy still provides employees with greater discretion and flexibility, its positive effects may be constrained when individuals feel less psychologically connected to their organization. In such situations, healthcare professionals may be less likely to translate autonomous work experiences into positive evaluations of their long-term career development (De Jong and Elfring, 2010). Furthermore, the significant quadratic and interaction effects observed in the nonlinear analysis suggest that the moderating role of organizational identification is not constant across all levels of job autonomy. Rather, the influence of organizational identification varies across different autonomy conditions, indicating a more complex and dynamic relationship than that implied by traditional linear moderation models (Bakker and Demerouti, 2017; Schaufeli et al., 2020). These findings support the view that organizational identification acts as a boundary condition influencing how healthcare professionals perceive and benefit from job autonomy in their career development process. Overall, the results highlight the importance of simultaneously promoting job autonomy and strengthening organizational identification in healthcare organizations. Enhancing both factors may create a more favorable environment for sustaining healthcare professionals’ career development satisfaction.

### 5.4 Research limitations and future directions

This study also has certain limitations and shortcomings. First, The cross-sectional study design can only reveal correlations between variables but cannot establish the direction of causality; for example, the bidirectional influence between job autonomy and career development satisfaction remains unclear. Second, the sample for this study was drawn from several public hospitals in Xuzhou, Jiangsu Province; therefore, the findings primarily reflect the characteristics of healthcare workers in the context of public hospitals in that region. There may be differences in the allocation of medical resources, organizational management models, and career development environments across different regions; thus, future research should validate these findings within a broader regional context. Third, the occupational composition of the sample was relatively unbalanced. Medical technicians constitute the majority of respondents, while nurses, clinicians, and primary-care health workers are underrepresented. This imbalance may limit the sample’s representativeness and affect the external validity of the findings. Future research should include a more diverse range of healthcare professions and institutions to enhance the generalizability of the results. Using the longitudinal tracking design, this study captures the dynamic evolution of the relationship among job autonomy, organizational identification, and career development satisfaction. Future research should integrate policy contexts, such as Diagnosis-Related Groups (DRG) payment reforms, to examine how external environmental factors may moderate the core relationships investigated in this study. Finally, this study identified a significant interaction effect between job autonomy and organizational identification. Future research could incorporate additional variables to enrich the examination of psychological mechanisms, thereby further exploring how job autonomy and organizational identification jointly influence career development satisfaction in career development.

## 6 Conclusion

This study examined the relationship among job autonomy, organizational identification, and career development satisfaction. The results indicate that job autonomy is a significant predictor of career development satisfaction, with the much stronger influence than other demographic and socioeconomic variables. In contrast, while organizational identification has the relatively weak direct effect on career development satisfaction, its interaction with job autonomy exhibits a significant moderating effect. Specifically, in contexts of low job autonomy, higher organizational identification can, to some extent, offset the negative effects of insufficient autonomy, thereby enhancing career development satisfaction; whereas in contexts of high job autonomy, the positive effect of organizational identification on career development satisfaction is weakened and may even exhibit a certain inhibitory effect. This suggests that career development satisfaction is not determined solely by a single factor but is rather the result of the dynamic interplay between job autonomy and organizational commitment. This study further enriches the empirical research on the relationship among job autonomy, organizational identification, and career development satisfaction, and demonstrates that organizational identification may play different roles at varying levels of job autonomy.

From the practical perspective, healthcare institutions should adopt differentiated management strategies based on specific job roles and work contexts. On the one hand, they should enhance healthcare professionals’ job autonomy by optimizing delegation mechanisms, expanding participation in clinical decision-making, and increasing autonomy in professional practice. On the other hand, for positions with relatively limited job autonomy, institutions should strengthen organizational support, foster a sense of shared values, and build organizational culture to enhance healthcare professionals’ organizational identification, thereby mitigating the potential negative impacts of insufficient autonomy. By simultaneously focusing on the synergistic effects of job autonomy and organizational identification, healthcare institutions can further enhance healthcare professionals’ satisfaction with their career development and promote the sustainable development of human resources.

## Data Availability

The original contributions presented in the study are included in the article/supplementary material, further inquiries can be directed to the corresponding author/s.

## References

[ref1] Ashforth B. E. Kreiner G. E. Fugate M. (2000). All in a day's work: boundaries and micro role transitions. Acad. Manag. Rev. 25, 472–491. doi: 10.5465/amr.2000.3363315

[ref2] Bakker A. B. Demerouti E. (2017). Job demands–resources theory: taking stock and looking forward. J. Occup. Health Psychol. 22, 273–285. doi: 10.1037/ocp0000056, 27732008

[ref3] Behera D. K. Padmaja M. Dash A. K. (2024). Socioeconomic determinants of happiness: empirical evidence from developed and developing countries. J. Behav. Exp. Econ. 109:102187. doi: 10.1016/j.socec.2024.102187

[ref4] Blau P. M. (2017). Exchange and Power in Social Life. New York: Routledge.

[ref5] Bogdán P. M. Zrínyi M. Madarász I. Tóth L. Pakai A. (2024). Work motivation: a wall that not even the COVID-19 pandemic could knock down. Healthcare 12:1857. doi: 10.3390/healthcare12181857 39337198 PMC11430960

[ref6] Burney I. A. Al Sabei S. D. Al-Rawajfah O. Labrague L. J. AbuAlrub R. (2023). Determinants of physicians’ job satisfaction: a national multi-Centre study from the Sultanate of Oman. Sultan Qaboos Univ. Med. J.. 23:198. doi: 10.18295/squmj.8.2022.050, 205, 37377833 PMC10292585

[ref7] Cziraki K. Wong C. Kerr M. Finegan J. (2020). Leader empowering behaviour: relationships with nurse and patient outcomes. Leadersh. Health Serv. 33, 397–415. doi: 10.1108/LHS-04-2020-0019, 33635019

[ref8] Dai Y. Tang Y. M. Chen W. Hou J. (2022). How organizational trust impacts organizational citizenship behavior: organizational identification and employee loyalty as mediators. Front. Psychol. 13:996962. doi: 10.3389/fpsyg.2022.996962, 36457918 PMC9706095

[ref9] De Jong B. A. Elfring T. (2010). How does trust affect the performance of ongoing teams? The mediating role of reflexivity, monitoring, and effort. Acad. Manag. J. 53, 535–549. doi: 10.5465/amj.2010.51468649

[ref10] Deci E. L. Olafsen A. H. Ryan R. M. (2017). Self-determination theory in work organizations: the state of a science. Annu. Rev. Organ. Psychol. Organ. Behav. 4, 19–43. doi: 10.1146/annurev-orgpsych-032516-113108

[ref11] Deci E. L. Ryan R. M. (2000). The" what" and" why" of goal pursuits: human needs and the self-determination of behavior. Psychol. Inq. 11, 227–268. doi: 10.1207/S15327965PLI1104_01

[ref12] Delic N. Djedovic I. Mekic E. (2021). The effects of autonomy on job satisfaction and job performance: evidence from Bosnia and Herzegovina. J. Hum. Res. Rehabil. 11, 126–132. doi: 10.21554/hrr.092109

[ref13] Diener E. Biswas-Diener R. (2022). Will money increase subjective well-being? Soc. Indic. Res. 57, 119–169. doi: 10.1023/A:1014411319119

[ref14] Dong R. Song L. Yang Y. Ni S. (2025). Job autonomy on psychological well-being: the mediating role of leisure activities. SAGE Open 15:21582440251357822. doi: 10.1177/21582440251357822

[ref15] Gagné M. Deci E. L. (2005). Self-determination theory and work motivation. J. Organ. Behav. 26, 331–362. doi: 10.1002/job.322

[ref16] General Office of the State Council (2021). Opinions on Promoting the high-Quality Development of public Hospitals (Guobanfa [2021] No. 18) Beijing General Office of the State Council. Available online at: https://www.gov.cn/zhengce/content/2021-06/04/content_5615473.htm (Accessed March 28, 2026).

[ref17] Gökhan A. B. A. Özkan Ş. Karçkay A. T. Çobanoğlu F. (2024). The relationship between job and career satisfaction among healthcare workers: the mediating effect of work-family conflict. Int. J. Disciplin. Econ. Admin. Sci. Stud. 8, 246–254. doi: 10.29228/ideas.57974

[ref18] Greenhaus J. H. Parasuraman S. Wormley W. M. (1990). Effects of race on organizational experiences, job performance evaluations, and career outcomes. Acad. Manag. J. 33, 64–86. doi: 10.5465/256352

[ref19] Gujarati D. N. (2003). Basic econometrics. 4th Edn. New York: McGraw-Hill.

[ref20] Hackman J. R. Oldham G. R. (1975). Development of the job diagnostic survey. J. Appl. Psychol. 60, 159–170. doi: 10.1037/h0076546

[ref21] Hair J. F. Black W. C. Babin B. J. Anderson R. E. (2010). Multivariate Data Analysis: A Global Perspective. 7th Edn New Jersey, NY: Pearson Education.

[ref22] Hajir-Afzali S. (2025). Autonomy as a double-edged resource: perceived organizational support, trust, and commitment in Korean hybrid work. Available online at: https://dqkx-periodicals.com/wp-content/uploads/2025/11/Hajir-Afzali.pdf (Accessed March 28, 2026).

[ref23] Hobfoll S. E. (1989). Conservation of resources: a new attempt at conceptualizing stress. Am. Psychol. 44, 513–524. doi: 10.1037/0003-066X.44.3.513, 2648906

[ref24] Hu Y. Tu W. Zhou L. Wu X. Yan Q. (2023). Evaluating emotional labor from a career management perspective. Front. Psychol. 13:1093723. doi: 10.3389/fpsyg.2022.1093723, 36726510 PMC9886059

[ref25] Ijarew (2022) The role of autonomous motivation and organizational culture on the relationship between job autonomy and organizational commitment. Available online at: http://ijeais.org/wp-content/uploads/2022/3/IJAMR220314.pdf (Accessed March 29, 2026).

[ref26] Jeon S. Mann A. Denis V. Hooley T. (2024). Challenging social inequality through career guidance: insights from international data and practice. Paris: OECD Publishing. doi: 10.1787/619667e2-en

[ref27] Ji D. Cui L. (2021). Relationship between Total rewards perceptions and work engagement among Chinese kindergarten teachers: organizational identification as a mediator. Front. Psychol. 12:648729. doi: 10.3389/fpsyg.2021.648729, 33995206 PMC8116523

[ref28] Kim T. T. Karatepe O. M. (2023). Outcomes of servant leadership among flight attendants: test of parallel and serial multiple mediating effects. Int. J. Contemp. Hosp. Manag. 35, 848–870. doi: 10.1108/IJCHM-02-2022-0156

[ref29] Klein M. (2019). Self-determination theory: basic psychological needs in motivation, development, and wellness. Sociol. Cas. 55, 412–413. Available online at: https://sreview.soc.cas.cz/pdfs/csr/2019/03/12.pdf

[ref30] Letouche S. Wille B. (2022). Connecting the dots: exploring psychological network analysis as a tool for analyzing organizational survey data. Front. Psychol. 13:838093. doi: 10.3389/fpsyg.2022.838093, 35592177 PMC9110883

[ref31] Li H. (2025). The relationship between innovation-oriented values and organizational commitment among new generation employees: the mediating role of work autonomy. Knowl. Econ. 17:133-135, 169. Available online at: https://www.ncpssd.cn/Literature/articleinfo?id=ZSJJ2025017041&synUpdateType=&type=journalArticle&typename=%E4%B8%AD%E6%96%87%E6%9C%9F%E5%88%8A%E6%96%87%E7%AB%A0&nav=1&langType=1&from=Qikan_Article_Detail

[ref32] Li M. Fan W. Zhang L. (2023). Career adaptability and career choice satisfaction: roles of career self-efficacy and socioeconomic status. Career Dev. Q. 71, 300–314. doi: 10.1002/cdq.12334

[ref33] Li J. Li S. Jing T. Bai M. Zhang Z. Liang H. (2022). Psychological safety and affective commitment among Chinese hospital staff: the mediating roles of job satisfaction and job burnout. Psychol. Res. Behav. Manag. 15, 1573–1585. doi: 10.2147/PRBM.S365311, 35769176 PMC9236165

[ref34] Li G. Wang Y. Deng C. (2025). Mechanisms of boundaryless career support on career development satisfaction and role performance. Chin. J. Manag. 22, 287–296. doi: 10.3969/j.issn.1672-884x.2025.02.009

[ref35] Liu X. Qian H. Gong X. Zhang Y. Yun Y. Yan J. . (2023). Influence of occupational stress on mental health of medical staff: mediating effect of affective commitment and moderating effect of overcommitment. J. Environ. Occup. Med. 40, 304–309.

[ref36] Lu J. G. Brockner J. Vardi Y. Weitz E. (2017). The dark side of experiencing job autonomy: unethical behavior. J. Exp. Soc. Psychol. 73, 222–234. doi: 10.1016/j.jesp.2017.05.007

[ref37] Mael F. Ashforth B. E. (1992). Alumni and their alma mater: a partial test of the reformulated model of organizational identification. J. Organ. Behav. 13, 103–123. doi: 10.1002/job.4030130202

[ref38] Maisonneuve F. Galy A. Groulx P. Chênevert D. Grady C. Coderre-Ball A. M. (2025). Managing resilience and exhaustion among health care workers through psychological self-care: the impact of job autonomy in interaction with role overload. J. Healthc. Leadersh. 17, 63–73. doi: 10.2147/JHL.S501193, 40034466 PMC11874763

[ref39] Maslach C. Schaufeli W. B. Leiter M. P. (2001). Job burnout. Annu. Rev. Psychol. 52, 397–422. doi: 10.1146/annurev.psych.52.1.397, 11148311

[ref40] Medeni V. Medeni İ. Altunay G. Dikmen A. U. İlhan M. N. (2025). Job satisfaction, life satisfaction, and associated factors among hospital nurses: a cross-sectional study in Türkiye. Sci. Rep. 15:5738. doi: 10.1038/s41598-025-85564-4, 39962150 PMC11832888

[ref41] Meyer J. P. Allen N. J. (1991). A three-component conceptualization of organizational commitment. Hum. Resour. Manag. Rev. 1, 61–89. doi: 10.1016/1053-4822(91)90011-Z

[ref42] Meyer J. P. Stanley D. J. Herscovitch L. Topolnytsky L. (2002). Affective, continuance, and normative commitment to the organization: a meta-analysis of antecedents, correlates, and consequences. J. Vocat. Behav. 61, 20–52. doi: 10.1006/jvbe.2001.1842

[ref43] Millar R. Chen Y. Wang M. Fang L. Liu J. Xuan Z. . (2017). It's all about the money? A qualitative study of healthcare worker motivation in urban China. Int. J. Equity Health 16:120. doi: 10.1186/s12939-017-0616-9, 28687089 PMC5501304

[ref44] Moon H. Jung Y. B. Han S. J. (2025). Comparative analysis of job satisfaction and determinants between medical and surgical hospitalists in South Korea: a nationwide cross-sectional online survey. Ann. Surg. Treat. Res. 109, 401–407. doi: 10.4174/astr.2025.109.6.401, 41368340 PMC12685455

[ref45] National Health Commission of the People's Republic of China (2025) 2024 China Health Statistical Yearbook Beijing National Health Commission. Available online at: https://www.nhc.gov.cn/guihuaxxs/c100133/202512/f1c3a3c617484a27a1a26a468afbaeee.shtml (Accessed March 29, 2026).

[ref46] Neveu J. P. Khan R. Murtaza G. (2024). Investing in resources: an interaction model of personal resources, commitment, and work achievement. J. Pers. 92, 361–377. doi: 10.1111/jopy.12826, 36810634

[ref47] Reza A. Anindita R. (2021). The importance of job autonomy as a driver of organizational commitment through work-life balance for employees in a life insurance company. Jurnal Aplikasi Bisnis Dan Manajemen (JABM) 7:614. doi: 10.17358/jabm.7.3.614

[ref48] Riketta M. (2016). “Organizational identification,” in Handbook of Employee Commitment, (Cheltenham, UK: Edward Elgar Publishing), 106–118.

[ref49] Romadhona A. A. Murti B. (2024) Associations between age, education level, and job satisfaction in health workers: a Meta-analysis. In The International Conference on Public Health Proceeding. 9 1 82–82

[ref50] Schaufeli W. B. Desart S. De Witte H. (2020). Burnout assessment tool (BAT)—development, validity, and reliability. Int. J. Environ. Res. Public Health 17:9495. doi: 10.3390/ijerph17249495, 33352940 PMC7766078

[ref51] Sen P. K. (1968). Estimates of the regression coefficient based on Kendall's tau. J. Am. Stat. Assoc. 63, 1379–1389. doi: 10.1080/01621459.1968.10480934

[ref52] Stinglhamber F. Marique G. Caesens G. Desmette D. Hansez I. Hanin D. . (2015). Employees' organizational identification and affective organizational commitment: an integrative approach. PLoS One 10:e0123955. doi: 10.1371/journal.pone.0123955, 25875086 PMC4395289

[ref53] Thapa D. R. Nunstedt H. Larsson I. Hallgren J. Ahlstrand I. Pennbrant S. (2025). Tasks contributing to job satisfaction among health professionals: a qualitative descriptive study. Nurs. Open 12:e70338. doi: 10.1002/nop2.70338, 41105165 PMC12533499

[ref54] van Dorssen-Boog P. van Vuuren T. de Jong J. Veld M. (2022). Healthcare workers' autonomy: testing the reciprocal relationship between job autonomy and self-leadership and moderating role of need for job autonomy. J. Health Organ. Manag. 36, 212–231. doi: 10.1108/JHOM-04-2022-0106, 36135716 PMC10424641

[ref55] Vander Weerdt C. Porter T. H. Peck J. A. (2026). Stress and coping in behavioral healthcare: a qualitative study. J. Health Organ. Manag. 40, 218–236. doi: 10.1108/JHOM-12-2024-0504, 40785635

[ref56] Wassermann M. Fujishiro K. Hoppe A. (2017). The effect of perceived overqualification on job satisfaction and career satisfaction among immigrants: does host national identity matter? Int. J. Intercult. Relat. 61, 77–87. doi: 10.1016/j.ijintrel.2017.09.001, 29527078 PMC5839331

[ref57] Wu M. Wang W. Chen W. Qiu H. (2026). Mediating effect of psychological capital between perceived organizational support and work engagement in Chinese nurses: a systematic review and meta-analytic structural equation modeling. Psychol. Health Med., 1–20. doi: 10.1080/13548506.2026.2628982, 41679748

[ref58] Zhao J. Liu T. Liu Y. (2024). Leadership support and satisfaction of healthcare professionals in China’s leading hospitals: a cross-sectional study. BMC Health Serv. Res. 24:1016. doi: 10.1186/s12913-024-11449-3, 39223660 PMC11370056

[ref59] Zhao S. Wang T. Luo S. Mi Y. Wang Y. Ma H. . (2025). The impact of organizational commitment on job performance in primary healthcare: a motivation internalization perspective. Front. Public Health 13:1685420. doi: 10.3389/fpubh.2025.1685420, 41103462 PMC12521417

[ref60] Zhou H. Long L. (2004). Statistical remedies for common method biases. Adv. Psychol. Sci. 12, 942–950. doi: 10.3969/j.issn.1671-3710.2004.06.018

[ref61] Zhu W. Li J. Wu L. Du F. Zhou Y. Diao K. . (2024). A latent profile analysis of doctors’ joy in work at public hospitals. Front. Psychol. 15:1330078. doi: 10.3389/fpsyg.2024.1330078, 38577117 PMC10991811

[ref62] Zito M. Colombo L. Borgogni L. Callea A. Cenciotti R. Ingusci E. . (2019). The nature of job crafting: positive and negative relations with job satisfaction and work-family conflict. Int. J. Environ. Res. Public Health 16:1176. doi: 10.3390/ijerph16071176, 30986910 PMC6480208

